# Role of the interstitium during septic shock: a key to the understanding of fluid dynamics?

**DOI:** 10.1186/s40560-023-00694-z

**Published:** 2023-10-10

**Authors:** Auguste Dargent, Hugo Dumargne, Marie Labruyère, Stéphane Brezillon, Sylvie Brassart-Pasco, Mathieu Blot, Pierre-Emmanuel Charles, Isabelle Fournel, Jean-Pierre Quenot, Marine Jacquier

**Affiliations:** 1 Service d’Anesthésie Médecine Intensive-Réanimation, Hospices Civils de Lyon, Hôpital Lyon Sud, 165 Chemin du Grand Revoyet, Pierre-Bénite, 69495 Lyon, France; 2 APCSe VetAgro Sup UPSP 2016.A101, 1 Avenue Bourgelat, 69280 Marcy l’Etoile, France; 3 Médecine Intensive et Réanimation, CHU François Mitterrand, 14 Rue Paul Gaffarel, 21000 Dijon, France; 4 UMR CNRS/URCA 7369, MEDyC, 51 Rue Cognacq-Jay, 51096 Reims, France; 5 Maladies Infectieuses et Tropicales, CHU François Mitterrand, 14 Rue Paul Gaffarel, 21000 Dijon, France; 6 Lipness Team, INSERM LNC-UMR1231 et LabEx LipSTIC, Université de Bourgogne, 7 Bd Jeanne d’Arc, 21000 Dijon, France; 7 Module Épidémiologie Clinique, Inserm, CHU Dijon, Bourgogne, Université de Bourgogne, CIC1432, 14 Rue Paul Gaffarel, 21000 Dijon, France

**Keywords:** Interstitium, Septic shock, Microcirculation, Capillary leak, Extracellular matrix

## Abstract

**Background:**

While not traditionally included in the conceptual understanding of circulation, the interstitium plays a critical role in maintaining fluid homeostasis. Fluid balance regulation is a critical aspect of septic shock, with a well-known association between fluid balance and outcome. The regulation of transcapillary flow is the first key to understand fluid homeostasis during sepsis.

**Main text:**

Capillary permeability is increased during sepsis, and was classically considered to be necessary and sufficient to explain the increase of capillary filtration during inflammation. However, on the other side of the endothelial wall, the interstitium may play an even greater role to drive capillary leak. Indeed, the interstitial extracellular matrix forms a complex gel-like structure embedded in a collagen skeleton, and has the ability to directly attract intravascular fluid by decreasing its hydrostatic pressure. Thus, interstitium is not a mere passive reservoir, as was long thought, but is probably major determinant of fluid balance regulation during sepsis. Up to this date though, the role of the interstitium during sepsis and septic shock has been largely overlooked. A comprehensive vision of the interstitium may enlight our understanding of septic shock pathophysiology. Overall, we have identified five potential intersections between septic shock pathophysiology and the interstitium: 1. increase of oedema formation, interacting with organ function and metabolites diffusion; 2. interstitial pressure regulation, increasing transcapillary flow; 3. alteration of the extracellular matrix; 4. interstitial secretion of inflammatory mediators; 5. decrease of lymphatic outflow.

**Conclusions:**

We aimed at reviewing the literature and summarizing the current knowledge along these specific axes, as well as methodological aspects related to interstitium exploration.

## Background

**Fig. 1:**
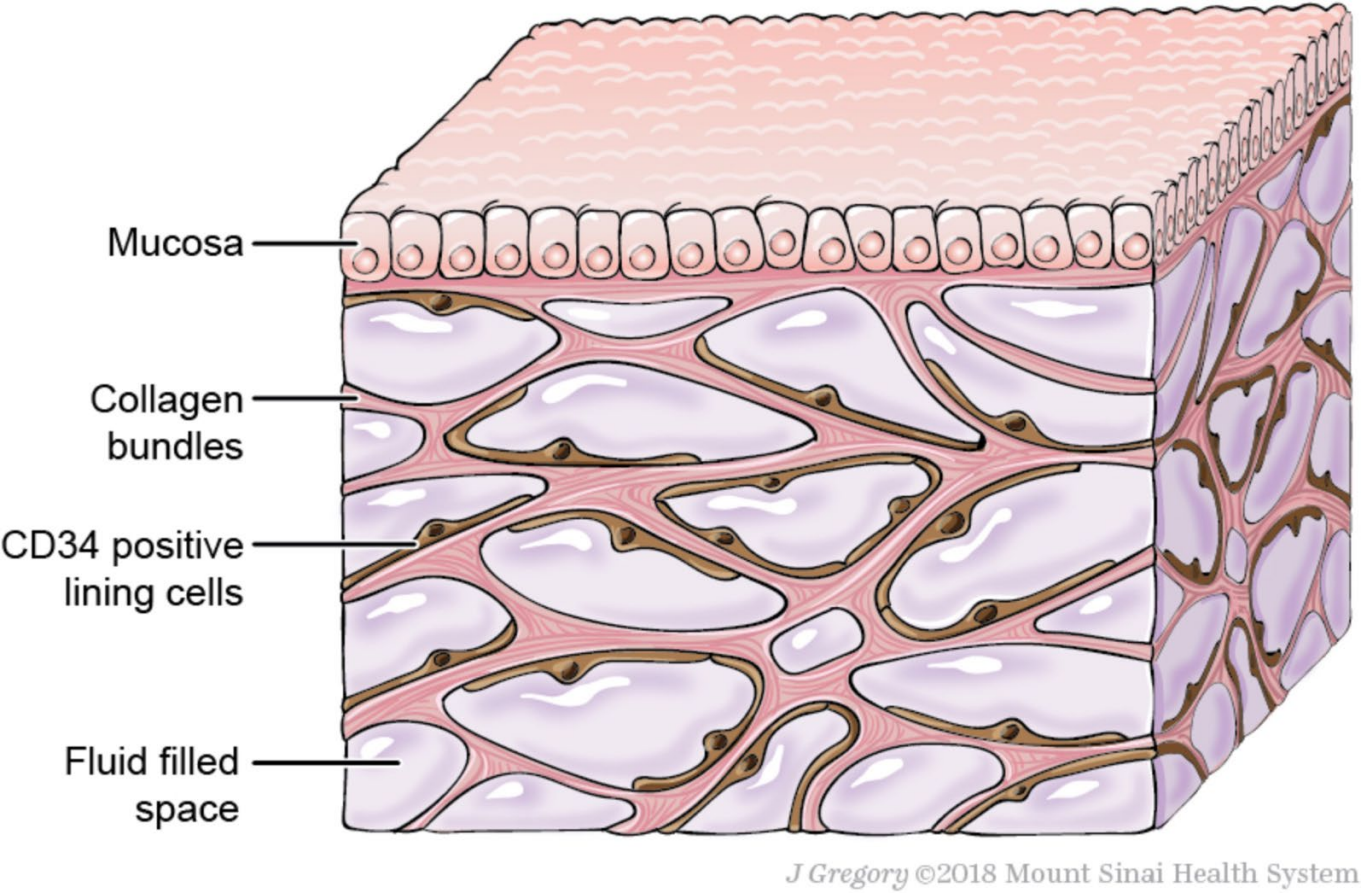
Three-dimensional structure of the interstitium. Schematic of the fluid-filled space supported by a network of collagen bundles lined on one side with cells. Illustration by Jill Gregory. Printed with permission from Mount Sinai Health System, licenced under CC–BY–ND. (https://creativecommons.org/licenses/by-nd/4.0/legalcode)

Interstitium can be defined as the interstitial, often virtual space existing between cells of any given tissue, but it is also a structured tissue in its own right. Advances in in vivo microscopy allowed the description of large anatomical spaces with thick collagen bundles delimiting fluid-filled polygons (Fig. 1), especially developed in the subcutis and the digestive tract submucosae [1]. Comprising around 20% of body weight [2], interstitium is mostly referred to as a fluid compartment, counterpart of the vascular compartment, from which it is separated by the capillary wall. Interstitium is not traditionally included in the conceptual understanding of circulation. However, between the cardiovascular and lymphatic system, interstitium is the essential link in the continuous fluid circulation that maintains homeostasis through the whole body (conveying metabolic substrates, waste products and immune mediators). The continuous transfer of fluids between the blood and interstitial compartment is governed by the so-called Starling forces. During sepsis and septic shock, acute changes in Starling forces lead to intense capillary leakage [3], responsible for the hypovolemia which characterizes the early phase of septic shock [4]. The development of diffuse oedema, a common feature of the later phase of septic shock, is also directly related to capillary leakage [5], although this relationship is masked by the delayed clinical recognition of oedema. Sepsis-induced oedema is aggravated by fluid therapy and is mostly referred to as fluid overload. It has been identified in recent years as a major prognostic factor for morbidity and mortality in patients with septic shock [6, 7].

As septic shock patients can gain up to one-third of their weight in a few days due to interstitium expansion, this “third space” deserves closer attention and should be understood as a whole by the intensivist. The interstitium has long been neglected and considered as a passive reservoir, whereas the vascular compartment was the only focus. Major physiology discoveries were made in the past decades, revealing how the interstitium plays a major role during acute inflammation, especially in regulating capillary filtration [8].

**Fig. 2:**
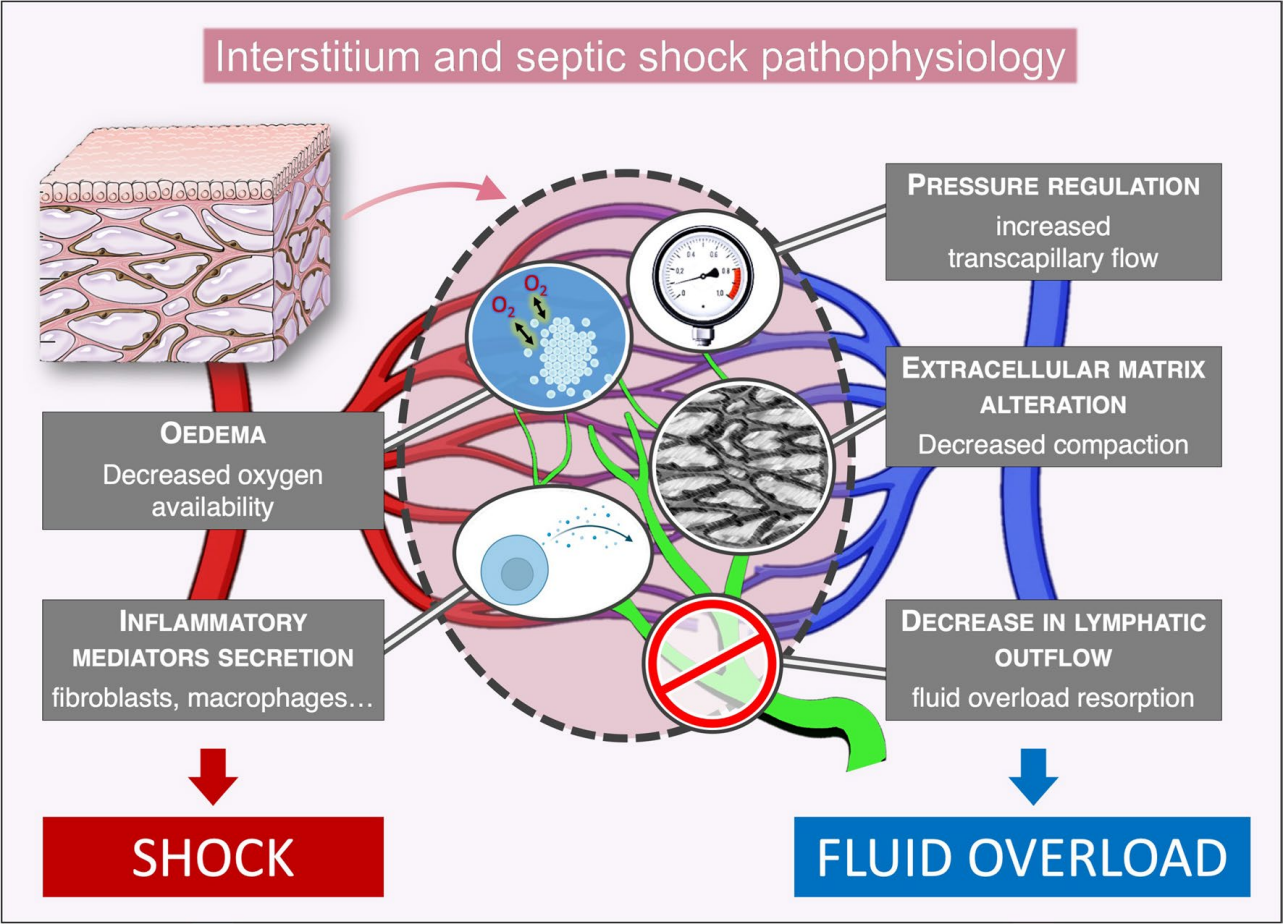
Proposed mechanisms by which interstitium may interact with sepsis pathophysiology. Diagram representing the interstitial compartment, surrounding the capillary bed (red: arterial side, blue: venous side). Lymphatic vessels appear in green

Overall, we have identified five potential intersections between septic shock pathophysiology and interstitium (Fig. 2): 1. increase of oedema formation, interacting with organ function and metabolites diffusion; 2. interstitial pressure self-regulation, increasing transcapillary flow; 3. alteration of the extracellular matrix (ECM); 4. interstitial secretion of inflammatory mediators; 5. decrease of lymphatic outflow.

To shed light on the potential role of the interstitium in the pathophysiology of septic shock, we aimed at reviewing the literature and summarizing the current knowledge along these specific axes, as well as methodological aspects related to interstitium exploration.

## Interstitium anatomy and histology

The interstitium is traditionally defined as a fluid compartment, separating blood vessels and cells. It was recently shown that the interstitial space was continuous across tissue and organ boundaries, allowing movement of particles between layers of the colonic wall or through subcutis and fascia [9]. An interstitial tissue is found in every organ, but its extent varies a lot between different “mother” tissues. The structure and composition of the interstitium may also vary, but remains similar in the interstitia surrounding peripheral blood vessels and loose connective tissue associated with dermal and glandular basal membranes [10]. Although interstitium composition has been known for long, its three-dimensional structure was only observed recently, especially thanks to the progress of in vivo microscopy (Fig. 1) [1]. Typically, the extracellular matrix (ECM) is scaffolded by a three-dimensional fibrous collagen network, in which collagen fibres form thick, 20 μm wide bundles. A microfibril–elastin framework is also attached to collagen bundles, uniformly distributing the stress to the structure [11]. The ground substance is formed from proteoglycans, composed from glycosaminoglycans (GAGs) covalently linked to a core protein, but also the free GAG hyaluronan. GAGs are long, linear structures comprising repeated disaccharide units. They are highly polar and attract water, forming gel-like structures. GAGs interact with the collagen structure of the ECM but are also found unbound in the interstitial fluid. The interstitial fluid itself is an ultrafiltrate from plasma with about 50% of its protein concentration [8]. The ECM is lined and supported by mesenchymal cells [1].

## Oedema formation: genesis and consequences of acute interstitial oedema

When inflammation is present in a circumscribed area, vascular flow and permeability are regionally increased to enable rapid recruitment of humoral (e.g., antibodies, complement) and cellular elements (neutrophils, monocytes), required to control the pathogens. During sepsis, global hyperactivation of innate immunity results in diffuse endothelial alterations leading to macro- and micro-circulatory dysfunction. Endothelial barrier alteration is probably the most relevant consequence at the capillary level, with impairment of each strata of the endothelial wall, enabling capillary leak [12]. VE-cadherin is a major component of endothelial intercellular junctions. Its internalisation, prompted by inflammatory cytokines (TNF-α, IL-1β, IL-6, and IL-10) is alone sufficient to break down intercellular junctions, thereby enhancing vascular permeability [13, 14], which is also influenced by other factors, such as glycocalyx alterations [15, 16]. Vascular permeability is often cited as both necessary and sufficient to drive and sustain capillary leak during septic shock. However, it should be kept in mind that other “forces” are at play and drive sepsis-associated fluid overload.

### Starling principles

The pathophysiology of capillary filtration, and thereby of oedema formation was first described by Ernest Henry Starling in 1896 [17]. Transcapillary filtration occurs as a function of various factors expressed in the following formula:

$${J}_{v}=Kf\cdot \left(\left[{P}_{c}-{P}_{i}\right]-\sigma \left[{\pi }_{c}-{\pi }_{i}\right]\right)=Kf\cdot \Delta Pf$$

where:

*Jv* is the trans-endothelial filtration volume per second; *Kf* is the coefficient of filtration of the membrane, *Pc* is the capillary hydrostatic pressure, *Pi* is the interstitial hydrostatic pressure, *πc* is capillary colloid osmotic pressure (COP), *πi* is the interstitial colloid osmotic pressure, σ is the reflection coefficient for plasma proteins, and Δ*Pf* is the net filtration pressure.

Capillary hydrostatic pressure declines, from around 30–40 mmHg at the end of the arterioles to 10–15 mmHg at the venular end. Plasma and interstitial oncotic pressure are, respectively, around 28 and 8 mmHg [18]. Normal interstitial pressure is slightly negative, at between − 1 and − 3 mmHg [19]. The classic view of the Starling principle predicted that absorption of fluid (from interstitium to vascular lumen) should occur at the venular end of the capillary, due to the drop in *P*_*c*_ and *π*_*i*_ at this point, and compensate in part the filtrated volume [20]. This classical view was revised by Levick and Michel in 2010, based on extensive prior experimental data, and leading to the “no steady-state filtration” rule [21]. Indeed, they showed that that *π*_*i*_ could be replaced in the classic Starling formula by sub-glycocalyx COP (*π*_*g*_), considering that the protein-reflecting element in the endothelial barrier was actually the glycocalyx fibre-matrix, acting like an ultrafiltrating sieve [22]. Due to the high velocity flow in intercellular clefts, interstitial proteins cannot diffuse back through the subglycocalyx space, and *π*_*g*_ remains lower than *π*_*i*_ (around 10%) but constant [21], whereas the classical view considered interstitial pressures as negligible. This explains the absence of observed filtration despite P_c_ decreasing below π_c_ in venules at steady state [23]. However, transient absorption can still occur, e.g., during haemorrhage, due to dramatic decrease in Pc and increasing π_c_. Absorption is also naturally occurring in intestinal mucosa and renal peritubular interstitium [24].

### Oedema preventing factors

**Fig. 3:**
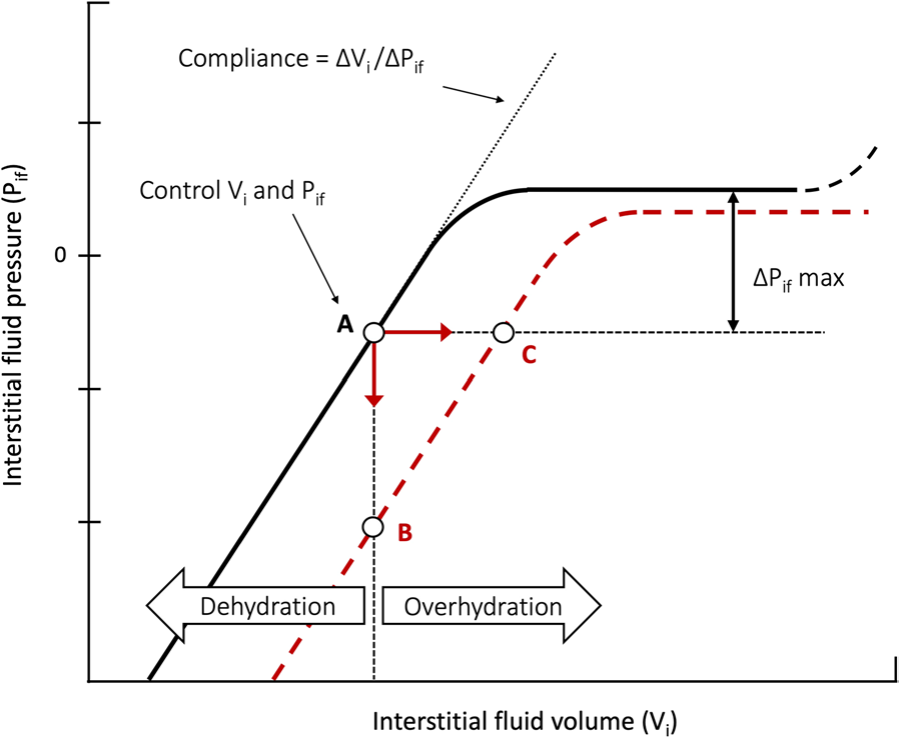
Interstitium pressure–volume relationship during homeostasis and inflammation. General shape of interstitial volume–pressure relationship (solid line) with normal values for interstitial volume (V_i_) and pressure (P_if_) (**A**). Compliance (∆V_i_/∆P_if_) is constant in dehydration and in the initial part of overhydration. Compliance then increases to infinity (plateau in the P/V curve), after a maximal rise in P_if_ (∆P_if_ max) is obtained (reflecting maximal counterpressure toward filtration). Compliance decreases again at excessive increase of interstitial fluid volume [2]. During inflammation, this relationship shifts (red dashed line). If filtration is impeded (thus preventing an increase in volume), e.g., by capillary flow interruption, P_if_ falls abruptly (**B**). If capillary flow is maintained, filtration rate may increase 10–20-fold and V_i_ can expand rapidly with little change in pressure (**C**) [116]

Thus, the revised Starling principle states that lymphatic drainage equals transcapillary filtration at steady state. Oedema formation is then due to an imbalance between increased capillary filtration and/or lowered lymphatic outflow. In physiology, the mechanisms protecting against oedema have been abundantly described, notably by Guyton [19]. First, an increase in capillary filtration augments the interstitial volume by reducing interstitial oncotic pressure by dilution, which in turn reduces filtration [21]. Another protective factor against oedema formation is interstitial compliance, defined as the variation of interstitial pressure (ΔPif) obtained with a given increase in interstitial volume (ΔVi) [25], and which is specific to each tissue. In a baseline state of dehydration and in the initial phase of overhydration, the shape of the volume–pressure curve is linear, which protects the tissue against oedema formation (Fig. 3, solid line). When there is extreme overhydration, compliance is potentially infinite, and a plateau of the volume–pressure curve is observed in skin and muscle interstitium [26]. The subcutaneous interstitial tissue is especially compliant and can accumulate a large volume of oedema [8]. During fluid overload, the exact contribution of the subcutaneous interstitium to the total volume is difficult to assess, but it could be above 50%, as skin and muscle already accounts, during steady state, for two-thirds of the total extracellular volume [26].

### Fluid overload, an expression of capillary leak and prognostic factor during septic shock

During initial management of septic shock, high volumes of resuscitation fluids are often needed to compensate for the capillary leak [27]. Oedema accumulates in all tissues, especially subcutaneous tissue [28, 29], and reflects the intensity of capillary leak. The development of oedema (i.e., fluid overload) has been identified in recent years as a major prognostic factor for morbidity and mortality in patients with septic shock [6, 7]. Yet, the nature of the link between fluid overload and poor outcome is not yet fully understood. First, it can be argued that the intensity of capillary leak at the acute phase is directly related to the severity of shock [5]. However, in most of the numerous studies finding this association, mortality was adjusted on initial shock severity [30]. In addition, observational studies in patients with septic shock determined that patients with negative fluid balance had better outcomes [31, 32], suggesting that interstitial oedema may cause harm in itself. Interstitial oedema increases intercellular spaces and has long been recognized as a critical factor for tissue oxygenation [33]. Indeed, the dioxygen molecule (O_2_) is an “hydrophobic”, nonpolar molecule, with low solubility and diffusivity in water, which makes its diffusion through aqueous media such as interstitial fluid and cytoplasm a challenge. It is thought that oxygen diffusion is helped through “hydrophobic channeling” via networked lipids, especially for intracellular diffusion [34]. Thus, interstitial oedema with increased diffusion distance could indeed be a prominent factor in the sepsis-associated decreased oxygen extraction and impaired metabolism [35]. Another link between interstitial oedema and outcome can be found in the direct contribution of oedema to organ dysfunction, as seen in acute respiratory distress syndrome (ARDS) [36], but also in acute kidney injury [37]. Some authors also described that fluid overload is a risk factor for the development of abdominal compartment syndrome [38].

In the light of these data, the reduction of fluid overload has become a primary therapeutic target. Fluid restriction is currently the main candidate therapeutic strategy aimed at reducing fluid overload. A meta-analysis of studies published between 2015 and 2020 did not find any significant benefit, whether on all-cause mortality or secondary outcomes, such as acute kidney injury or lung injury [39]. In 2022, Meyhoff and al. reported the results of the CLASSIC trial, which also did not find any difference in mortality (nor any secondary outcomes) between a restrictive and a liberal fluid strategy [40]. The CLOVERS trial, published in 2023, confirmed these findings with no difference in mortality, and an increased vasopressor use in the restrictive group with (non-significantly) increased adverse events [41]. Overall, the effect of the intervention on fluid balance was low and not always statistically significant. The negative results of these trials underline the complexity of this issue, and suggest that the approach of fluid restriction is probably not appropriate.

### Interstitial fluid assessment: interstitial volume

Oedema begins to form very early, but becomes apparent only 24–48 h after the onset of septic shock, as it is not clinically detectable below approximately 4 L [14]. Indeed, clinical appraisal of fluid overload is insufficient in septic shock and lacks sensitivity. Daily fluid balance monitoring is the simplest, most straightforward way to measure the extent of fluid overload. In a historic cohort of septic shock patients, Boyd reported a cumulative average fluid balance of + 11 L by day 4, a more positive fluid balance being associated with an increased risk of mortality in these patients [42]. The most accurate measurement of the blood compartment volume and transcapillary filtration requires the injection of a bolus of albumin labeled with radioactive iodine, allowing the measurement of its transcapillary escape rate (TER). This technique was used to evaluate the benefit of albumin infusions during sepsis, which failed to decrease vascular permeability [43]. In a randomized trial using isotopic blood volume and TER analysis in critically ill patients (with a majority of septic shock), fluid management was changed by the analysis in 44% of cases, and the overall mortality was significantly decreased in the intervention arm [44]. This underpins the difficulty to assess the fluid status of critically ill patients. The discrepancy between intravascular and interstitial volume was elegantly explored with a “volume kinetics” method, using the haemodilution induced by an infusion of crystalloids [45]. This method was able to predict the repartition of fluids in the plasma and the interstitium in healthy volunteers and anesthetized patients, and also confirmed the “interstitial albumin washout” phenomenon predicted by Guyton [45].

## Interstitial pressure (and transcapillary flow) regulation during inflammation

### Interstitial pressure measurement

Arthur C. Guyton pioneered the exploration of the interstitium, and developed the reference technique for measuring interstitial pressure with the implantation of a perforated capsule in animal models [46]. The pressure could be measured after allowing several weeks for healing and stabilization. In the 1980s, many new approaches emerged to measure interstitial pressure. Various subcutaneous catheter techniques were then developed in animal models, with either fluid-filled side ported catheters or using the wick-in-needle technique [47]. Since then, most experimental studies [48, 49] have used glass micropipettes linked to an automated counterpressure system. Finally, miniaturization of pressure sensors has enabled the development of reliable and accurate catheters for measuring pressure, although their size remains significantly greater than that of micropipettes [50].

### Discovery of interstitial pressure regulation in models of burn injury

If a rise in P_if_ can prevent oedema formation, a decrease in P_if_ is sufficient to enhance transcapillary filtration and promote oedema formation. In healthy volunteers, the application of negative pressure on the lower limbs led to an almost instantaneous drop in interstitial pressure in the leg with increased fluid filtration and leg oedema [51]. During inflammation, the rapid formation of oedema is facilitated not only by changes in the endothelial barrier, but also a sudden drop in interstitial pressure [52]. Indeed, in the case of a burn, a visible oedema appears within a few minutes. The interstitial volume must at least double for an oedema to become visible [48]. The filtration rate must, therefore, increase several hundred times above the normal to generate oedema in such a short time, since the interstitial fluid usually turns over in 12 to 24 h. Studies measured Kf variations during experimental burn injury, which was increased by a factor of 2 to 3 [53, 54]. With this modest increase in Kf, it was calculated that the net filtration pressure would have to increase to 200 mmHg to explain the observed filtration rate [53]. To explore this hypothesis, Lund et al*.* measured interstitial pressure in an experimental model of thermal burn injury in rats. They observed that intradermal P_if_ was reduced from normal level of − 1 mmHg to highly negative values (− 150 mmHg) after thermal injury [52]. The hypothesis of an increase in net filtration pressure was thus confirmed, mainly driven by a reduction in P_if_, rather than an increase in P_c_. This landmark study, whose results were subsequently confirmed by other teams in similar models [55], was the first to demonstrate an active role (via “aspiration”) for the interstitial tissue.

### Extension to other local and systemic inflammation models

The phenomenon of “interstitial suction” has been reproduced in models of systemic inflammation, such as during anaphylactic reaction induced in rats by the administration of intravenous dextran [56], with a reduction of − 10 mmHg in P_if_ in subcutaneous interstitial tissue. Similarly, “septic” inflammation seems to bring on the same effects. In an experimental rat model, injection of LPS reduced interstitial pressure and contributed to oedema formation with extravasation of I-125-marked albumin [57]. A comparable reduction was previously observed in a model of endotoxic shock in dogs, with an immediate reduction in interstitial pressure of up to 9 mmHg [58].

Apart from inflammation, the power of this interstitial “suction” phenomenon is also illustrated in the salivary glands, where it is allowing the bursts of salivary secretion which require, in response to a vegetative stimulus, an increase in the transcapillary flow by a factor of ten [59]. This is a striking illustration on how this phenomenon, in a daily physiologic function, has the ability to recruit a large volume from the circulation, in a limited time.

Interestingly, it was found that adrenergic vasoconstriction and vasopressors also had a lowering effect on P_if_. In their study, Border et al. explored the effect of different shock types and isolated vasopressors on interstitial fluid pressure measured with the Guyton technique. As mentioned earlier, endotoxic shock was characterized by a marked, although transient lowering of P_if_ (increasing V_i_ due to capillary filtration explains the return to pre-shock values). However, in hemorrhagic shock and with the isolated perfusion of catecholamines, P_if_ was also lowered, but in a constant and dose-dependent manner [58]. This was predicted, according to authors, by the Starling principles, as the vascular volume is decreased by vasopressors (thereby increasing relative V_i_). These results could also be explained by a decreased capillary flow due to extreme vasoconstriction [60].

### Role of the extracellular matrix and fibroblasts

Oedema is, therefore, not accidental, but rather represents a finely tuned mechanism and necessary component of the local immune response (by facilitating the trafficking of humoral and cell mediators). Indeed, the phenomenon of “interstitial suction” contributes, as seen above, to inflammatory oedema. The regulation of interstitial pressure is primarily based on the interaction between fibroblasts and the ECM.

**Fig. 4:**
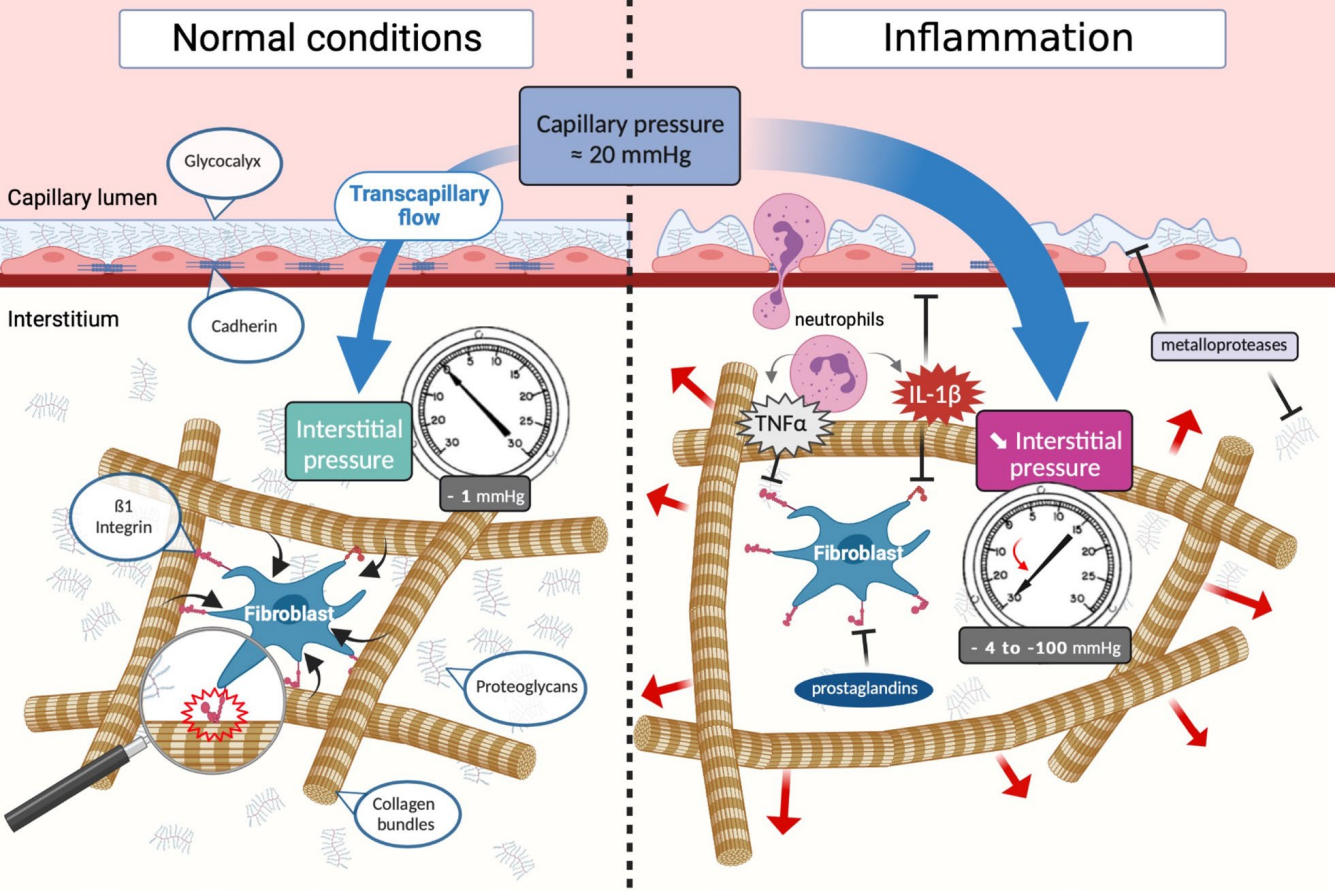
Interaction between the microcirculation and interstitial extracellular matrix during homeostasis and sepsis. In steady state, transcapillary flow is regulated by a solid endothelial barrier and a compacted interstitium with stable, slightly negative pressure (left panel). Fibroblasts apply constant tension to the network of collagen bundles. Fibroblasts’ cytoskeleton is tethered to collagen IV through transmembrane protein ß1 integrin. When inflammation is present (right panel), the binding of collagen bundles by ß1 integrin is inhibited by inflammatory mediators (especially IL-1ß), releasing the physical tension constraining the collagen network, which leads to an abrupt decrease in interstitial pressure [10]. Furthermore, like the glycocalyx, the extracellular matrix is altered by the various proteases released by innate immune cells. The massive increase of transcapillary flow is allowed by the resultant increase in filtration pressure (capillary pressure—interstitial pressure) and by a parallel increase in endothelial permeability due to intercellular adhesion inhibition and glycocalyx shedding

Fibroblasts interact with the ECM and play a role in regulating interstitial pressure within this molecular network. They express specific integrins that are capable of binding to collagen fibres, notably via their ß1 subunit (e.g., integrin α2ß1). Integrins are transmembrane proteins comprising 2 subunits that enable bidirectional adhesion, attaching the cell cytoskeleton to the ECM. The ß1 integrin subunit, therefore, enables transmission of constant mechanical tension to the ECM via the interaction with collagens, opposing the natural tendency of GAGs to expand with hydration. Thus, a sudden depression can be caused by rapid release of the pressure exerted on the collagen fibres (Fig. 4) [10].

The role of ß1 integrin subunit in the formation of oedema has been demonstrated in vitro by the assessment of fibroblast-mediated contraction of floating collagen type I gels [61]. In this study, it was shown that fibroblasts are capable of contracting collagen gels to 10% of the initial gel volume within a 24-h incubation period. These findings were confirmed by in vivo experiments in which blockade of ß1-integrin adhesion receptors in rat skin led to a decrease in interstitial pressure and formation of oedema [49, 62]. During inflammation, pro-inflammatory mediators such as IL-1β and TNF-α counteract this contraction via their activity on the β1 integrin subunit, causing a reduction in interstitial pressure, and subsequently, oedema [63]. The platelet-derived growth factor (PDGF) exerts an inverse effect on this cascade linked to the β1 integrin, leading to an increase in interstitial pressure via the contraction of fibroblasts and the mediation of another integrin subunit (αVβ3) [64]. In tumors, where interstitium is enlarged and interstitial pressure is increased, PDGF inhibitors reverse the tumor-induced increase in interstitial pressure, and improve antitumor response by promoting capillary filtration and local diffusion of antitumor agents [65].

Furthermore, ECM’s GAGs are also involved in interstitial volume regulation. They are negatively charged and interact in particular with sodium, the most abundant cation in the extracellular fluid. Depending on their spatial conformation, GAGs accumulate a varying quantity of sodium, which can contribute to variations in interstitial volume, oedema formation and resolution [66, 67]. During sepsis, sodium load is increased due to hyperaldosteronism, a phenomenon that is thus enhanced by the ECM’s “hunger” for sodium, which could overall hinder oedema resolution and interfere with the complex interaction between interstitial sodium, macrophages osmosensors and VEGF-induced lymphangiogenesis [67].

## Extracellular matrix alteration

The ECM forms the physical structure of the interstitial space. We described the functional changes induced by inflammation, leading to ECM decompaction. Moreover, inflammation also leads to the direct alteration of ECM structural compounds due to the release by infiltrating neutrophils of potent catalytic enzymes, such as MMPs (matrix metalloproteases), heparanases and hyaluronidases [68], activated by IL-1β and TNF-α [69]. During sepsis as well, evidence was found of massive ECM degradation, giving rise to the concept of “systemic wound” [70]. In septic shock patients at the time of diagnosis, circulating levels of cross-linked type I collagen telopeptides (ICTP), a marker of collagen I degradation, were five times higher compared to controls [70]. In this study, markers of collagen synthesis were also increased, although it was only the case for the precursor of collagen III and not collagen I. Levels of ICTP and procollagen were also higher in non-survivors compared to survivors [70]. Increased levels of ICTP were also found in patients with Gram-negative sepsis [71], and increased procollagen III levels were found in other studies in septic patients [72, 73]. Procollagen III is also increased during ARDS, and might be an important contributor to the progression to fibrosis [74].

A small descriptive study from Koskela and collaborators [75], reported that the epidermal expression of both laminin-332 and type IV collagen was decreased early in severe sepsis, and for up to 3 months in survivors. Hoffmann and collaborators reported elevated levels of matrix metalloproteases and their inhibitors (MMP-9, TIMP-2 and TIMP-1) in severe sepsis, and TIMP-1 was suggested as a useful biomarker in predicting the clinical outcome of patients with severe sepsis [76]. The prognostic role of TIMP1 and MMP9 during sepsis was confirmed by another study [77].

Other ECM macromolecules, such as proteoglycans and small leucine-rich proteoglycans (SLRP), can be synthesized de novo or released from collagen association during ECM remodelling. Recently, Maiti G and collaborators reported that during sepsis, lumican (a proteoglycan from the SLRP family) is endocytosed by immune cells and is able to control receptor ligand trafficking [78]. Lumican promoted TLR4 but restricted TLR9 during sepsis: it may thus play a dual protective role in barrier ECM tissues, by promoting bacterial defense and control antiviral and autoimmune inflammatory responses [78]. Circulating GAGs were also used to monitor inflammation-induced ECM alteration during disease, like in rheumatoid arthritis [79], and acute pancreatitis [80]. Elevated levels of circulating GAGs were also described during sepsis, mostly as a marker of the degradation of the endothelial glycocalyx, a thick layer of glycosaminoglycans covering the endoluminal side of the endothelium, comprising a network of glycosaminoglycans and proteoglycans anchored to the endothelial cell wall [81]. Animal models of endotoxemia demonstrate the sepsis-induced shedding of the glycocalyx, with an increased plasma concentration of its byproducts (e.g., syndecan-1 and heparin sulfate) [82], in response to the same enzymes as the ECM [15]. Median plasma levels of glycosaminoglycans (GAGs) resulting from vascular damage, were found to be increased almost fourfold in septic shock patients, and correlate with shock severity and mortality rate [83, 84]. The major role of glycocalyx in sepsis pathophysiology is now well-known. However, glycocalyx is considered to be a cellular counterpart, an extension or even a specialized form of ECM [85]. Indeed, their structure and composition are very similar, although glycocalyx does not include collagen fibers but hyaluronan and transmembrane proteoglycans, such as syndecan. Glycocalyx is in fact an external organelle for every cell, serving as a physical interface with the ECM (or blood in the case of the thicker endothelial glycocalyx) [86].

The consequences of ECM alteration during sepsis are not known yet. It could participate in the increased capillary filtration, as ECM physical structure is a key to the regulation of interstitial pressure: collagen and proteoglycans alteration could enhance the “decompaction” of the ECM. This is supported by experimental data: enzymatic digestion potentiates the ex vivo swelling of loose connective tissue immersed in isotonic saline, which is normally restrained by the collagen fibers network [87]. Furthermore, the collagen-protecting role of vitamin C was proposed as a potential mechanism to explain its oedema preventing effect in a model of burn injury [88]. Moreover, the alteration of the ECM may be especially relevant in the process of oedema resorption, i.e., the de-escalation phase of the septic shock fluid management. First, the altered ECM may have lost its ability to increase interstitial pressure and initiate a pressure gradient for lymphatic drainage, as compared with an intact ECM. Second, the decrease of the collagen in the interstitium increases the interstitial protein content, due to the phenomenon of steric exclusion by the collagen fibres, described by Wiig et al*.* [89]. Indeed, with a normal ECM, the apparent distribution volume of albumin and other macromolecules is lower than the total interstitial volume, because the multiple spaces delimited by the collagen network are not fully available to these molecules. An increased interstitial protein mass can increase colloid osmotic pressure and directly impair fluid transfers, as an higher lymph flow (“washout”) will be required to restore interstitial fluid volume [2]. Although clinical data are lacking yet, healing of the ECM may be a prerequisite for achieving negative fluid balance in septic shock patients.

### Extracellular matrix repair and adaptation

Increased interstitial flow has biophysical effects on ECM and residing cells. Mechanical stress, as well as inflammation, can induce the differentiation of fibroblast into myofibroblasts, and may ultimately lead to fibrogenesis [90]. It was found that increased interstitial flow could induce fibroblast motility through MMP-1 upregulation [91] as well as drive myofibroblast differentiation and matrix alignment, in a TGF-ß-dependent mechanism [92]. The alignment of the ECM was observed 12–24 h after flow onset, whereas myofibroblast differentiation occurred after 1–5 days. Interestingly, ECM’s fibers reorganize perpendicularly to the flow, decreasing the matrix’s conductivity. Another interesting effect of this realignment is a change in the shear stress repartition, transferred away from the cells and onto the matrix fibers [93]. Matrix alignment and fibroblast contraction also increase the ECM stiffness. Overall, these in vitro findings suggest that persistent increased interstitial flow can rapidly lead to ECM stiffening, alignment and myofibroblast differentiation, which are hallmarks of interstitial fibrosis.

It is yet unknown if interstitial fibrosis can develop in critically ill patients with persistent inflammation, but it might then contribute to inadequate healing with a persistent state of fluid overload, as seen in the more severe patients.

## Interstitium-derived inflammation (local production of mediators)

### Interstitial fluid assessment: collection and analysis of interstitial fluid

In the view of the emerging role of the interstitium, it appears that interstitial fluid is a promising medium for the discovery of new biomarkers. However, access to this fluid is difficult. In the 1900s, metal tubes were introduced subcutaneously for the evacuation of oedema in patients with heart failure (“Southey’s tubes”) [94]. This invasive technique was abandoned, but we can still observe in ICU that subcutaneous interstitial fluid is easily flowing through any cutaneous effraction in patients with overt oedema. The technique of pre-nodal lymphatic cannulation is probably the best way to obtain “pure” interstitial fluid [95], but it is invasive and requires microsurgery skills. Microdialysis was developed to determine the biochemical composition of interstitial tissue [96]. It is now mainly used for the monitoring of critically ill patients with brain injury to identify ischemia-associated metabolic changes [97]. Due to the semi-permeable membrane, it is limited to the analysis of ions and small metabolites. The microperfusion technique does not involve an exchange membrane and, therefore, enables analysis of a wider range of molecules, such as cytokines. It is widely used in pharmacologic studies, especially for topical drugs [98].

### Interstitial exploration during sepsis and other inflammatory diseases

The interstitial compartment is much more difficult to access than the blood for the analysis of biomarkers. However, numerous markers of inflammation are of local origin. Olszewski et al. performed lower leg lymphatic cannulation in patients with rheumatoid arthritis and in control subjects and collected prenodal lymph continuously for 72 h [99]. A lymph/serum ratio greater than 1, indicating local production, was found for most of the pro-inflammatory cytokines measured (IL-1 β, TNF-α, IL-6, IL-8). Those cytokines are also primarily involved in sepsis pathophysiology and produced for the most part by macrophages [100]. Furthermore, the interindividual variations in cytokine concentration, or the variations induced by treatment with methylprednisolone, were clearly visible at the lymphatic level, whereas the serum concentration did not vary significantly [99].

Using a microperfusion catheter inserted into the abdominal cutaneous adipose tissue of nine patients with severe sepsis, Ikeoka et al. found that the concentrations of IL-1β, IL-6 and IL-8 were higher in subcutaneous adipose tissue than in serum, indicating a subcutaneous interstitial production of these pro-inflammatory mediators, remotely from the infection site [101]. In addition, blood pressure was negatively correlated with subcutaneous concentrations of IL-1β, IL-6 and IL-8. Subcutaneous interstitial production of IL-1 has previously been demonstrated in a rat model of endotoxaemia using skin centrifugation [102], suggesting that the subcutaneous interstitium is involved during a systemic inflammatory stimulus. Immunohistochemical studies showed that the cells at the origin of this production of cytokines were primarily interstitial fibroblasts, but also epidermal cells and hair follicles [102]. The same authors have shown a direct mechanistic role of IL-1β and TNF-α concentrations in the formation of inflammatory oedema, and reported an immediate drop in interstitial pressure after exposure of subcutaneous tissue to similar cytokine concentrations as those found in endotoxaemia [63] or in models of ischemia–reperfusion [103]. Remote interstitial production of cytokines was also demonstrated in the subcutaneous adipose tissue of patients undergoing cardiac surgery, with increased IL-6 in the interstitial fluid, originating from the adipocytes with nuclear factor-κB-regulated genes activation [104]. The interstitial production of cytokines may also find its origin in a contingent of CD34 + (a pan-myeloid marker, also present at the surface of certain mesenchymal cells) interstitial fibroblasts observed in the microscopic study of sub epithelial interstitium by Benias et al. [1]. Indeed, CD34 fibroblasts secrete large amounts of IL-6, CXCL12, and CCL2 when stimulated with tumor necrosis factor (TNF) in vitro, suggesting their role in the recruitment of monocytes during inflammation [105].

## Alteration of lymphatic outflow

The lymphatic system plays a critical role in the circulation. At steady state, pre-nodal lymph flow equals capillary filtration, with an estimated 8–12 L reabsorbed daily through the lymphatic system [106], beginning in the interstitium as blind-ended sacs. The junctions between endothelial cells function as one-way valves for interstitial fluid, and drain into the precollecting lymphatics. Subatmospheric pressures are maintained by the rhythmic contraction of smooth muscle cells enclosed in the walls of lymphatic vessels, along with unidirectional valves [107]. Like blood vessels, lymphatic vessels respond to inflammatory mediators. Thus, lymphatic contractility is down-regulated by classic vasodilator mediators, such as NO, prostaglandins [108], histamine [109], and also with proinflammatory cytokines [110]. Lymphatic drainage is yet very important for the trafficking of antigens and dendritic cells to the lymph nodes. This explains the intense, CD11 + macrophages-induced lymphangiogenesis observed during local inflammation, which compensates the decrease in lymphatic contractility and allows an increase in regional lymph flow [111]. On the contrary, during acute conditions with systemic inflammation, such as sepsis, lymphangiogenesis may not be sufficient to overcome the global effect of inflammation-induced lymphatic relaxation. Thereby, inflammation-induced decrease in lymphatic drainage may be a major contributor to the deregulation of fluid balance during sepsis, and was considered by some authors as a therapeutic target [112].

Moreover, lymphatic alterations are also critical in the development of organ damage, and may prevent healing mechanisms. In the lungs, the role of lymphatic circulation is evidenced in lung transplantation, during which lymphatic vessels are interrupted. Lung transplant recipients exhibit persistent oedema, and the role of lymphatic vessels and lymphangiogenesis is suspected in primary graft dysfunction [113]. Resorption of pulmonary oedema plays a critical role for the outcome of ARDS. Fluid from the alveolar lumen is transported to the interstitium via transmembrane pumps. In LPS-induced acute lung damage, improved fluid clearance and survival was linked to increased lymphangiogenesis markers [114]. The role of lymphatics in acute organ failure was also evidenced in a murine model of acute kidney injury (AKI), where expansion of lymphatics improved recovery, suggesting an important role of lymphangiogenesis to prevent the progression of AKI to chronic kidney disease [115]. Although clinical data are lacking in human sepsis, the role of lymphatic drainage is obviously very important especially in the resolution phase of sepsis, both for organ dysfunction and general fluid overload resorption.

## Conclusions

The interstitium is not merely a passive bystander but finally emerges as a key player in the regulation of transcapillary flow, in particular with its ability to aspirate fluid from the blood vessels. This important role in the regulation of capillary filtration is increasingly well-documented in the setting of local inflammation and in tumours, but evidence of interstitium involvement during sepsis is still scarce. A lot of questions remain open as to its exact role during sepsis: what is the magnitude of pressure changes? Are there organ-specific responses? What is the respective role of subcutaneous fibroblasts and adipocytes in cytokine secretion? What is the role of ECM damage on oedema formation and resolution? In the current era of personalized medicine, the study of this little-known compartment could yield valuable insights, enhance our understanding of the pathophysiology and open unique perspectives for the investigation of new biomarkers and therapies.

## Data Availability

Not applicable.

## Abbreviations

ARDS: Acute respiratory distress syndrome
COP: Colloid osmotic pressure
∆P_f_: Net filtration pressure
ECM: Extracellular matrix
GAG: Glycosaminoglycan
ICTP: Cross-linked type I collagen telopeptides
J_v_: Trans-endothelial filtration volume per second
K_f_: Coefficient of filtration
MMP: Matrix metalloprotease
P_c_: Capillary hydrostatic pressure
P_i_: Interstitial hydrostatic pressure
π_c_: Capillary colloid osmotic pressure
π_g_: Sub-glycocalyx colloid osmotic pressure
π_i_: Interstitial colloid osmotic pressure
PDGF: Platelet-derived growth factor
SLRP: Small leucine-rich proteoglycans
σ: Reflection coefficient for plasma proteins
TER: Transcapillary escape rate
